# Interpopulation variation of transposable elements of the *hAT* superfamily in *Drosophila willistoni* (Diptera: Drosophilidae): *in-situ* approach

**DOI:** 10.1590/1678-4685-GMB-2021-0287

**Published:** 2022-03-16

**Authors:** Natasha Ávila Bertocchi, Thays Duarte de Oliveira, Maríndia Deprá, Beatriz Goñi, Vera Lúcia S. Valente

**Affiliations:** 1 Universidade Federal do Rio Grande do Sul, Programa de Pós-Graduação em Genética e Biologia Molecular, Porto Alegre, RS, Brazil.; 2 Universidade Federal do Rio Grande do Sul, Programa de Pós-Graduação em Biologia Animal, Porto Alegre, RS, Brazil.; 3 Universidad de la República, Facultad de Ciencias, Montevideo, Uruguay.

**Keywords:** Transposable elements, Drosophila willistoni, *hAT* superfamily, polytene chromosomes

## Abstract

Transposable elements are abundant and dynamic part of the genome, influencing organisms in different ways through their presence or mobilization, or by acting directly on pre- and post-transcriptional regulatory regions. We compared and evaluated the presence, structure, and copy number of three *hAT* superfamily transposons (*hobo, BuT2*, and *mar*) in five strains of *Drosophila willistoni* species*.* These *D. willistoni* strains are of different geographical origins, sampled across the north-south occurrence of this species. We used sequenced clones of the *hAT* elements in fluorescence *in-situ* hybridizations in the polytene chromosomes of three strains of *D. willistoni*. We also analyzed the structural characteristics and number of copies of these *hAT* elements in the 10 currently available sequenced genomes of the *willistoni* group. We found that *hobo, BuT2,* and *mar* were widely distributed in *D. willistoni* polytene chromosomes and sequenced genomes of the *willistoni* group, except for *mar*, which is restricted to the subgroup *willistoni.* Furthermore, the elements *hobo, BuT2*, and *mar* have different evolutionary histories. The transposon differences among *D. willistoni* strains, such as variation in the number, structure, and chromosomal distribution of *hAT* transposons, could reflect the genomic and chromosomal plasticity of *D. willistoni* species in adapting to highly variable environments.

## Introduction

Transposable elements (TEs) constitute part of the repetitive fraction of the genome and can move within and between host genomes. TEs are thought to be present in virtually all genomes and are best studied in the genus *Drosophila* (Diptera: Drosophilidae) (Wicker et al., 2007). TEs are considered generators of evolutionary novelty, as they can interact with host genomes in a variety of ways, although they were previously characterized as junk DNA. They can be found close to regulatory regions over- or under-expressing genes, as constituents of heterochromatin, and may increase the propensity to chromosomal variations, among other possible roles such as an inducer of cancers (Cáceres et al., 2001; Catania et al., 2004; Bertocchi et al., 2018)

The proposed life cycle of TEs can be summarized as: insertion by Horizontal Transposon Transfer (HTT) or reactivation in the host genome; increase in copy number (proliferation) and dispersal in the host population; and, over time, accumulation of mutations (diversification) (Wallau et al., 2012). Sexual reproduction eventually allows TEs to be distributed in most individuals of a population and/or species. At any stage of the cycle, a TE can be lost by the genome or, to a lesser extent, undergo HTT and restart the cycle (Schaack et al., 2010; Wallau *et al.*, 2012). HTT has been shown to perpetuate TEs in host genomes, and HTT events are increasingly identified in the most varied groups of eukaryotes (Wells and Feschotte, 2020). 

As classified by Wicker et al. (2007), TEs are divided hierarchically, first into two classes according to the transposition mechanism: class I via intermediary RNA (retrotransposons) and class II via intermediary DNA (transposons). Class II elements, termed transposons, use the enzyme transposase for mobilization; they are subdivided into two subclasses according to the number of DNA strands that are cleaved in the transposition process. Subclass 1 elements cleave the two strands of DNA by a “cut-and-paste” mechanism, and subclass 2 elements cleave only one of the strands, which has other transposition mechanisms. TEs are then classified into orders, superfamilies, families, and subfamilies according to their structural characteristics and conservation of nucleotide and amino-acid sequences.

TEs may also be classified according to their autonomy for mobilization. TEs can be autonomous, that is, possess the entire enzymatic structure needed to carry out their own mobilization; or non-autonomous, when they need the enzymatic machinery of other autonomous TE copies to mobilize. An example of Class II non-autonomous elements are termed miniature inverted-repeat transposable elements (MITEs) (González and Petrov, 2009). MITEs are cross-mobilized by autonomous elements, as they generally conserve the recognition sequences for transposases, the TIRs. They can also be found in high copy numbers in genomes (Deprá et al., 2012; Loreto et al., 2018). 

The *hAT* superfamily is present in animals, plants, and fungi. It is subdivided into three families: *Ac*, *buster*, and *tip* (Arensburger et al., 2011; Zhang et al., 2013; Rossato et al., 2014). Elements of the *hAT* superfamily have an 8 bp target site duplication (TSD) and short Terminal Inverted Repeats (TIRs) between 10-25 bp and 2.5-5 kb in size (Feschotte and Pritham, 2007). The elements *hobo*, *BuT2*, and *mar* belong to the *Ac, tip*, and *buster* families respectively (Deprá et al., 2012; Rossato *et al.*, 2014). The canonical *hobo* (HFL1) was initially described in *Drosophila melanogaster* and consisted of 2959 bp length, encoding a 1.9-kb transposase gene, and 12 bp of TIRs (Calvi et al., 1991). *Hobo* was originally described to be limited to the *melanogaster* subgroup (Ortiz and Loreto, 2008; reviewed in Loreto *et al.*, 2018). *BuT2* is 2775 bp long and encodes a 643 aa transposase and 12 bp of TIRs (Rossato *et al.*, 2014). *BuT2* was initially described in *Drosophila buzzatii*, in regions of inversion breakpoints, which indicates a recent mobilization, although it is only sparsely present in the genome of this species (Cáceres et al., 2001; Casals et al., 2006). The canonical *mar*-MITE element was originally identified in *D. willistoni* and has 610 bp and 11 bp of TIRs. *Mar* is restricted to the *willistoni* subgroup, and until now partially complete copies have been found only in *Drosophila tropicalis*, with approximately 2600 bp (Holyoake and Kidwell, 2003; Deprá *et al.*, 2012).

Dobzhansky (1950) described the first polytene photomap of *D. willistoni,* this map was further redrawn in Valente and Araújo, 1985, Regner et al., 1996, Bhutkar et al., 2008, and Rohde and Valente, 2012 *.* The chromosome complement of *D. willistoni* consists of two pairs of metacentric chromosomes (IIL, IIR, XL, and XR arms), an acrocentric pair (III arm), and a Y submetacentric chromosome (Dobzhansky and Powell, 1975; Santos-Colares et al., 2003). *D. willistoni* is notable for having multiple chromosomal inversions in every natural population examined (review by Rohde and Valente, 2012).

The first sequenced genome of *D. willistoni* was that of strain Gd-H4-1, the result of several generations of sister-brother crosses to obtain a strain without segregating inversions, i.e., a monokaryotypic strain (Drosophila 12 Genomes Consortium *et al.*, 2007). Strain Gd-H4-1 lacks the high degree of polymorphism and variability found in natural populations of this widely distributed tropical species (review by Zanini et al., 2015). Two additional strains of *D. willistoni* were recently sequenced by Kim et al. (2021), who found considerable differences between these strains in the number of repetitive sequences such as transposons and microsatellite elements. 

Our research group has been studying several aspects of the chromosomal plasticity of *D. willistoni* (Valente and Araújo, 1985; Valente *et al.*, 1993; Valente *et al.*, 2003; Rohde and Valente, 2012; Garcia et al., 2015). The goal of the present study was to contribute to understanding the high degree of variability of *D. willistoni* over its wide geographical distribution. In view of the significant environmental differences encountered by this species, the chromosomal variations characteristic for *D. willistoni* strains, and the differences found in the number of repetitive fractions in different strains, we compared and characterized the organization and distribution of three transposable elements of the *hAT* superfamily in different *D. willistoni* strains. Studies such as this can clarify how different habitats are capable of promoting evolutionary changes in TEs and hosts.

## Material and Methods

### Fly stocks and chromosomal preparations

Three strains of *D. willistoni* were used in this study (Table S1). These strains have been maintained in the laboratory by mass crosses and cultivated in cornmeal culture medium (Marques et al., 1966) under controlled temperature (20 ± 1 °C). The polytene chromosome preparations were obtained with third-instar larval salivary glands, squashed, and fixed in 2:1:2 ethanol-lactic acid-acetic acid, v/v. 

### Probe preparation and fluorescence *in-situ* hybridization (FISH)

TE clones were used as a template for the PCR labeling probe for FISH: *BuT2* in *D. willistoni* (GenBank accession number KF669641.1) obtained from Rossato et al. (2014)⁠; *mar_trop*: sequence of the *mar* element of *D. tropicalis* (GenBank accession number JQ654772.1) (obtained from Deprá et al., 2012)⁠; and *hobo* in *D. willistoni* (submitted GenBank accession number OK032551, this study). For this last, genomic DNA from strain Gd-H4-1 was used to amplify the *hobo* transposon. The primers used were *hobo* CN 991 (5′-ACCGTCGACATGTGGAC-3′) and *hobo* CN 1598 (5′-GGATGGCAATAGGAAGC- 3′) (Deprá *et al.*, 2009). The amplified sample was visualized on 0.8% agarose gel. The bands were purified using the GFX Purification Kit (GE Healthcare) and cloned using the TOPO-TA cloning vector (Invitrogen, Carlsbad, CA, USA). Cloned PCR products were sequenced using the universal primers M13 (forward and reverse) at Macrogen (Korea). 

The TE probes *BuT2, mar_trop*, and *hobo* were marked directly by PCR, using Biotin-16-dUTP (Jena Bioscience). Slide preparations, hybridizations, and washes were performed according to Deprá et al. (2010), with minor modifications. FISH experiments were established in 77% of the stringency. The signal was detected using streptavidin-Cy3 and the chromosomes were counterstained with Fluoroshield with DAPI. The slides were analyzed using the epifluorescence microscope ZEISS Axiophot (Zeiss, Germany). The images were captured using Zeiss ZEN (blue edition) software. The final editing of the images used Adobe Photoshop CS6. The hybridization signals were quantified by visual inspections and using the ImageJ software (Schneider et al., 2012). The following premises were applied to measurements of the hybridization signals: area less than 9.99 µm^2^ as non-hybridization borderline, and an area larger than 10 µm^2^ for each hybridization on the five chromosome arms (XR, XL, IIR, IIL, and III). In the chromocenter, we considered the presence or absence of a hybridization signal. 

### Genome searches

Searches for homologous sequences to *BuT2*, *hobo*, and *mar* were carried out in the genomes of the species of the *willistoni* group available in NCBI and Kim et al. (2021), last accessed in January 2021. Versions of the assemblies, species, and strains used in this study are available in Table S2. 

The queries used were: *mar* sequence from *D. tropicalis* (GenBank accession number JQ654772.1), *BuT2* (GenBank accession number KF669641.1), and *hobo* (GenBank accession number OK032551) from *D. willistoni* available in NCBI. BLASTn searches were performed on the Galaxy platform, using default parameters (Afgan et al., 2016). The sequences with an E-value lower than e^-10^ were extracted for each genome. 

### Sequence analysis

The sequence alignments were performed using MAFFT (Katoh and Standley, 2013), with default parameter values. AliView (Larsson, 2014) was used for sequence editing and visualization. *Mar* sequences are very variable in copy number, length, and structure, and therefore the alignment for phylogenetic reconstruction of the *mar* copies was submitted to two refinement steps: 1) copies with 100% of the identity in each genome were filtered by the CD-HIT Suite (Huang et al., 2010); and subsequently, 2) the alignment was manually inspected to exclude small and/or very degenerate sequences. All *mar* sequences after refinement (MITEs, relics, complete and partially complete) were used in phylogenetic reconstruction, except: Dwil_Gd_scf2_3; Dins_ctg2309_5, Dins_ctg424, Dins_ctg1175, Dins_ctg1948; Dtro_ctg108_3, Dtro_ctg108_4, Dtro_ctg838, Dtro_ctg804, Dtro_ctg19. All sequences of *hobo* and *BuT2* retrieved were used in the phylogenetic trees. 

The phylogenetic trees were inferred by Bayesian Analysis in MrBayes 3.2.6. implemented in the CIPRES gateway (Miller et al., 2010; Ronquist et al., 2012). The evolutionary models GTR+G (*hobo*), HKY+G (*BuT2*), and JC+I (*mar*) were indicated by MrModeltest2 (Nylander, 2004). The analysis was run for at least 10,000,000 generations, sampling trees every 1,000 generations, with 25% of the initial results as burn-in. MEGAX (Kumar et al., 2018) was used to measure the divergence of the sequences by p-distance and Neighbor-Joining phylogenetic reconstruction for the *mar* sequences (data not shown). 

Also, we performed a phylogenetic reconstruction of the *D. willistoni hobo* in the *hAT* superfamily, using the Maximum Likelihood method and Le-Gascuel model (LG) (Le and Gascuel, 2008) by MEGA X (Kumar et al., 2018), with the transposase database based on Arensburger et al. (2011) and Rossato et al. (2014). The transposase sequences were aligned by MUSCLE, implemented in MEGA X. 

## Results

### FISH of the *BuT2*, *hobo*, and *mar* elements in polytene chromosomes of *D. willistoni* strains

**Figure 1 -:**
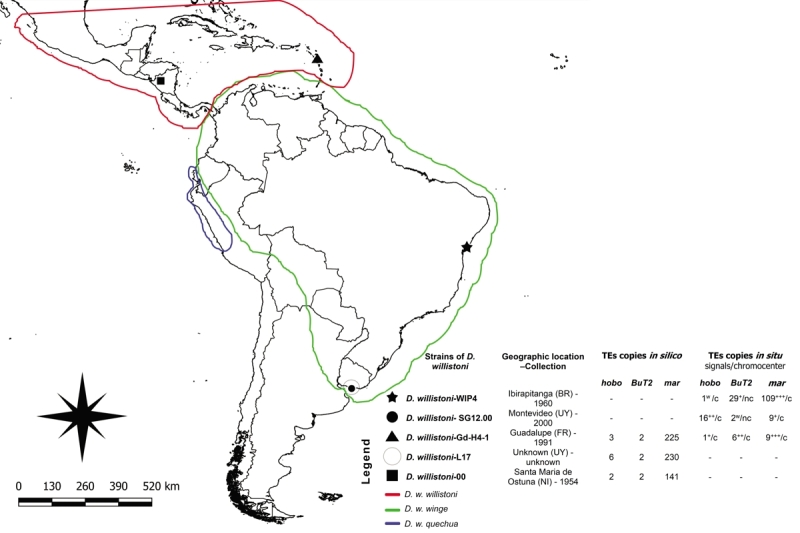
Geographical origins of the *Drosophila willistoni* strains analyzed *in silico* and *in situ,* and information about *hAT* TE copies. Lines indicate the approximate geographical distributions of the three subspecies of *Drosophila willistoni* (Mardiros et al., 2016). The numbers of TE copies in polytene chromosomes were measured by ImageJ software (Schneider et al., 2012) and visually. The ordinal number represents stronger signals, and increasing from + to +++ indicate the relative strength of intensity of signals on chromosome arms, as detected visually on polytene chromosome arms. In the table: - indicates absence of information; c and nc indicate presence and absence of signals on the chromocenter, respectively; w indicates weak signals; +,++ and +++, increasing from + to +++ indicate the relative strength of intensity of signals detected visually on polytene chromosomes by FISH.

**Figure 2 -:**
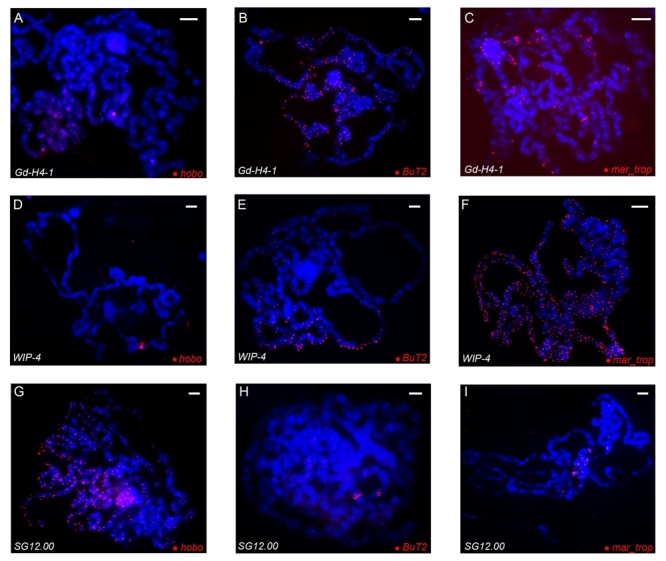
FISH in polytene chromosomes of *Drosophila willistoni* strains: (A-C) *D. willistoni-*Gd-H4-1; (D-F) *D.* *willistoni-*WIP-4; and (G-I) *D. willistoni-*SG12.00. The probes used are indicated in the lower right corner and the strains in the lower left corner of the images. Chromosomes were counterstained with DAPI (blue) and transposable element probes were labeled with Cy3 (red). Scale bar=10 µm.

For the FISH experiments, we used polytene chromosomes from the three strains of *D. willistoni* from different geographic locations. The strains were: *D. willistoni*-Gd-H4-1, an inbred lineage; *D. willistoni*-WIP-4, descended from a natural population maintained in the laboratory for approximately 60 years and considered by us a standard karyotype for the species; and a natural population, *D. willistoni*-SG12.00, collected in the 2000s in Montevideo (Figure 1 and Table S1). Clear differences were detected in the number and distribution of signals along the chromosomal arms of these strains (Figure 2A-I).

The three probes used were derived from clones of TEs *BuT2*, *hobo*, and *mar*, and were termed *BuT2*, *hobo*, and *mar_trop*, respectively. FISH experiments with the *BuT2* probe revealed differences among the strains in the distribution and number of signals. In *D. willistoni*-Gd-H4-1, visually many strong signals were detected along all chromosomes and the chromocenter (Figure 2B), while in *D. willistoni*-WIP-4 visually strong signals were observed on the IIR and IIL chromosome arms (Figure 2E). In *D. willistoni*-SG12.00, only two stronger signals of *BuT2* hybridization signals were visible on the IIR and IIL arms (Figure 2H), and some signals were detected also in the chromocenter. We noted a pattern in the production of signals according to the geographic origin of the strains; the northernmost strain (from above the Equator; Figure 1) had more signals and more intense signals than the other, more southern strains (Figure 1).

With the *hobo* probe, the pattern was almost the opposite of that seen for *BuT2*: the strain from the extreme southern part of the distribution (*D. willistoni*-SG12.00 - Figure 1) showed many stronger signals on all chromosome arms, mainly in the euchromatin and chromocenter (Figure 2G). *D. willistoni*-Gd-H4-1 and *D. willistoni*-WIP-4 showed one stronger signal on the IIR arm, and we also observed more signals with less intensity in the *D. willistoni* -Gd-H4-1 (Figure 2A, D).

Concerning the *mar_trop* probe, *D. willistoni*-WIP-4 showed many stronger signals in all chromosome arms and the chromocenter (Figure 2F). Although the ImageJ software estimated around the same number of *mar* copies in the strains *D. willistoni*-Gd-H4-1 and *D. willistoni*-SG12.00 (Figure 1), differences between the two strains were apparent (Figure 2C and 2I), mainly concerning the intensity and distribution of the signals along the chromosomal complement. *D. willistoni*-Gd-H4-1 showed stronger signals along the five chromosome arms and the chromocenter, while *D. willistoni*-SG12.00 showed signals on the chromocenter and on the arms near the chromocenter, with no signals observed on the III chromosome. 

### Transposons *in-silico* search in *Drosophila willistoni* group genomes

*hobo* search 

The cloned fragment of the element *hobo* from *D. willistoni*-Gd-H4-1 contained 439 bp and was 74.7% identical to that of the *D. melanogaster* canonical *hobo* (Calvi et al., 1991). *D. willistoni-hobo* alignments were mainly between nucleotide positions 991 and 1428 of the canonical *hobo* element. The BLASTn search showed that the *D. willistoni*-*hobo* fragment presented 93% identical to the *hobo* element of the Mediterranean fruit fly *Ceratitis capitata* (Diptera: Tephritidae) (Cc-HRE-GenBank access number U51454.1) (Handler and Gomez, 1996). To establish the relationship between the *D. willistoni-hobo* and *Cc-HRE* (*C. capitata)* putative transposase and the *hAT* superfamily elements, we assembled the transposase sequences described by Arensburger et al. (2011) and Rossato et al. (2014). A phylogenetic reconstruction of the *hAT* superfamily showed that *D. willistoni*-*hobo* and Cc-HRE (*C. capitata-hobo*) were grouped with *Ac* family elements (Figure S1). These formed a clade with *Howilli2* (*D. willistoni*), *Cc-HRE* (*Ceratitis capitata*), *Homo1* (*D. mojavensis*), canonical *hobo* (*D. melanogaster*)*, Hermes* (*Musca domestica*; Diptera: Muscidae), *Homer* (*Bactrocera tryoni*; Diptera: Tephritidae), *Hoana1* (*Drosophila ananassae*), *Hoana8* (*D. ananassae*), *Hermit* (*Lucilia cuprina*; Diptera: Calliphoridae), and *Hoana3* (*D. ananassae*).

**Figure 3 -:**
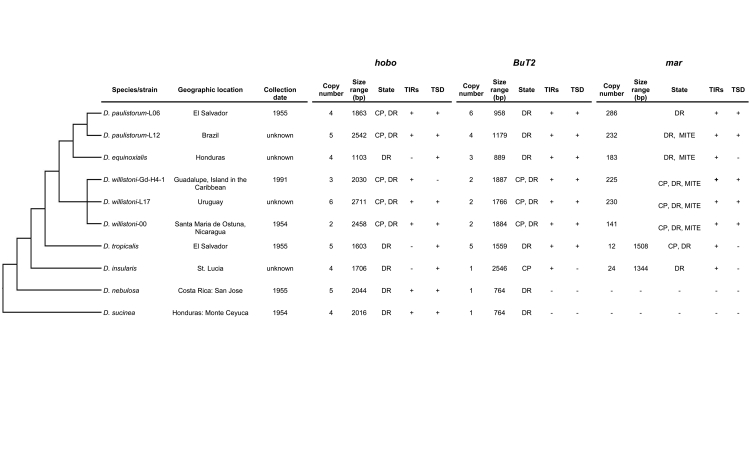
Information on species and evolutionary relationships of sequenced genomes of the *willistoni* group. Schematic evolutionary relationships among species of the *willistoni* group are based on Finet et al., (2021). (-) Absence; (+) presence; State = Structural characteristics of TE; CP = Complete or Partially complete copies; DR = Degenerate or Relic copies; MITE = miniature inverted-repeat transposable elements.

**Figure 4 -:**
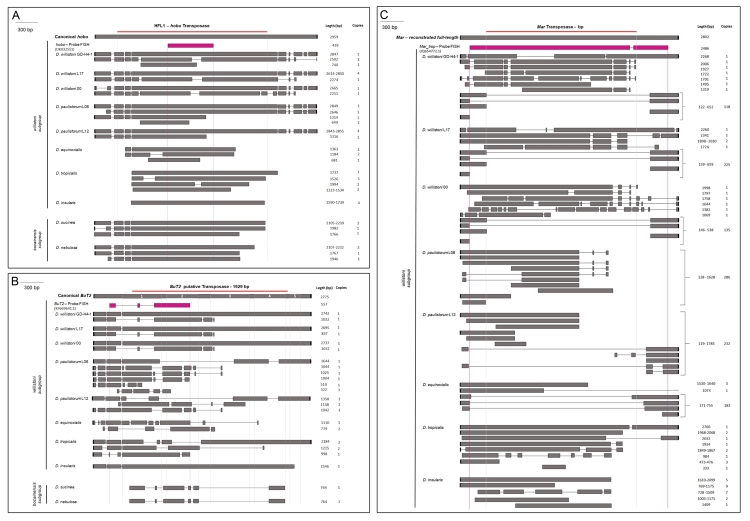
Schematic representation of reconstructed *hobo, BuT2*, and *mar* copies in the *willistoni* group. (A) *hobo:* all sequences are represented; (B) *BuT2:* all sequences are represented and transposase is formed by 5 exons, indicated by descending ordinal numbers; (C) *mar* : *mar-*MITE and degenerate sequences in *D. willistoni-*Gd-H4-1, *D. willistoni-*L17, *D. willistoni-*00, *D. paulistorum-*L06, *D. paulistorum*-L12, and *D. equinoxialis* were grouped. Regions of terminal inverted repeats shown inside black block, transposase coding region inside red line, and probes used in FISH experiments inside pink block. Only indels and deletions of nucleotides with more than 10 bp are represented**.**

Using the *hobo* sequence obtained here (cloned element from *D*. *willistoni* strain Gd-H4-1), we performed BLASTn against the 10 sequenced genomes belonging to seven species of the *willistoni* group (Table S3). Sequences homologous to the *hobo* fragment from *D. willistoni* were identified in the seven species of the *willistoni* group: *D. willistoni* (three strains), *D. paulistorum* (two strains), *D. equinoxialis*, *D. tropicalis*, *D. insularis, D. sucinea*, and *D. nebulosa* (Figure 3 and Figure 4A). In a search for complete copies of *hobo* in these genomes, we recovered homologous sequences and added 3000 bp from the *hobo* on each end. However, no complete copies were identified (codifying transposase and TIRs at the ends). A schematic representation of these sequences is shown in Figure 4A.

With respect to the *willistoni* subgroup, in the genomes of *D. willistoni-*Gd-H4-1, *D. willistoni-*L17, *D. willistoni-*00, *D. paulistorum-*L06*,* and *D. paulistorum-*L12 we identified the most complete copies of *hobo* (≈2850 bp), with small additions in the region of the transposase, 12 bp TIRs conserved and identical to the canonical *hobo* and TSDs (Figure 3, Figure 4A and Table S3). In the genomes of *D. willistoni-*Gd-H4-1, *D. paulistorum-*L06, and *D. paulistorum-*L12 we also observed smaller *hobo*-like fragments without TIRs at both ends (Figure 4A).

The *hobo*-like sequences retrieved from the *D. equinoxialis*, *D. tropicalis*, and *D. insularis* genomes are smaller fragments (Figure 3), conserved mainly in the 520 to 1720 bp region of canonical *hobo* transposase, without TIRs or conserved TSDs (Figure 4A and Table S3).

In the *bocainensis* subgroup, complete sequences of *D. sucinea* and *D. nebulosa* were not identified (Figure 3). Copy Dsuc_ctg141 in *D. sucinea* and copies Dneb_ctg3 and Dneb_ctg46 in *D. nebulosa* had identical canonical TIRs (Figure 4A and Table S4). In both species, TSDs were not present or were variable. 

**Figure 5 -:**
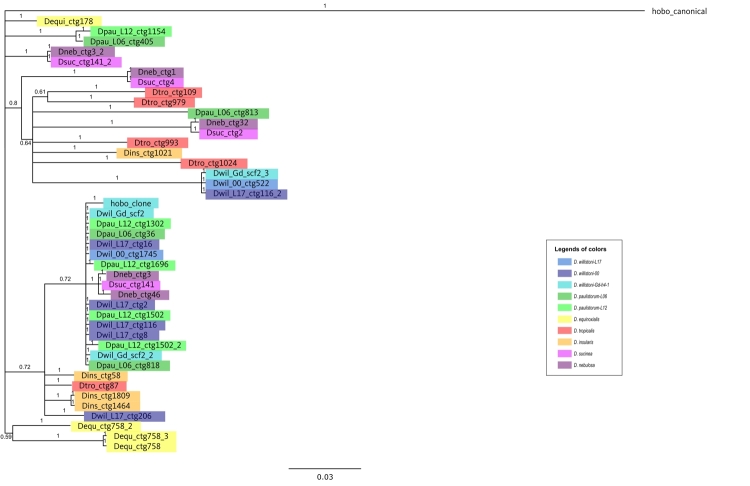
Phylogenetic relationships of *hobo* copies in the *willistoni* group. Unrooted Bayesian tree (GTR+G) based on nucleotide sequences. Node supports are shown by posterior probability. *Drosophila melanogaster hobo* canonical sequence is shown in black; the *hobo*_clone was used in the FISH experiments. Different strains and species are indicated in different colors, as shown in the legend. Further information on *hobo* sequences is available in Table S3.

In order to address the average divergence of the *hobo* sequences found within and between species/strains, we evaluated the p-distance (Table S4). The intragenomic divergences in *D. willistoni* strains were 3.91% in *D. willistoni-*L17, 13.64*% in D. willistoni-*00, and 13.88% in *D. willistoni-*Gd-H4-1. Intragenomic divergence of 4.1% was observed in *D. paulistorum-*L12 and 7.98% in *D. paulistorum-*L6. The values of interspecies divergence ranged from 3.74% between *D. paulistorum-*L12 and *D. willistoni-*L17 to 13.05% between *D. tropicalis* and *D. willistoni-*Gd-H4-1. Figure 5 shows the Bayesian tree obtained for all *hobo* copies from the *willistoni* species group identified in this study. The phylogeny showed low resolution in several nodes, groupings with sequences of different species and subgroups, and some polytomies. One group was formed by *D. willistoni* strains, *D. paulistorum* strains, *D. nebulosa*, and *D. sucinea* copies. The recurrent grouping between sequences of *D. nebulosa* and *D. sucinea* was also evidenced. The relationships among the *willistoni* group species together with the branch lengths indicate that these sequences are very similar, likely with recent mobilization. 

*BuT2* search

Sequences homologous to the element *BuT2* were detected in the 10 genomes of the *willistoni* group analyzed; however, no complete TE copies were identified (Figure 4B). In the subgroup *willistoni,* partially complete copies of *BuT2* were identified in *D. willistoni* strains. However, in the *bocainensis* subgroup (*D. sucinea* and *D. nebulosa*), only one short partial *BuT2* fragment (764 bp) without TIRs was identified in both species (Figure 3).

In *D. willistoni*, two homologous *BuT2* sequences were identified in the sequenced strains. The most complete sequences, i.e., in *D. willistoni-*Gd-H4-1 with 2742 bp (Dwil_scf2_2), *D. willistoni-*L17 with 2695 bp (Dwil_ctg8), and *D. willistoni-*00 with 2737 bp (Dwil_ctg1698), were 91% identical to the *BuT2* element including 12 bp TIRs (Figure 4B and Table S5). Furthermore, the Dwil_scf2_2, Dwil_ctg8, and Dwil_ctg1698 sequences of *BuT2* in *D. willistoni* were flanked by 8 bp TSDs. In these three *D. willistoni* strains, the TSDs were conserved, with one mismatch in *D. willistoni*-Gd-H4-1 (Table S5). The other copies of *D. willistoni-*Gd-H4-1 (Dwil_scf2), *D. willistoni*-L17 (Dwil_ctg326), and *D. willistoni-*00 (Dwil_ctg675) were incomplete: without the TE initial region, with a size of 837-1032 bp, TIRs conserved in the TE 3’ region, and with large deletions in the exon regions 1, 2 and 4 of transposase (Figure 4B).

*BuT2* copies in *D. paulistorum*-L06, *D. paulistorum*-L12, *D. equinoxialis*, and *D. tropicalis* genomes were defective, with deletions in the five exons of *BuT2* transposase (Figure 3, Figure 4B, and Table S5). The 12 bp TIRs were conserved in *D. paulistorum-*L06, *D. paulistorum-*L12, and *D. tropicalis* copies (Figure 3 and Table S5). The *D. insularis* genome with one *BuT2* copy lacked the 263 bp 5’ end of TE (Figure 4B).

**Figure 6 -:**
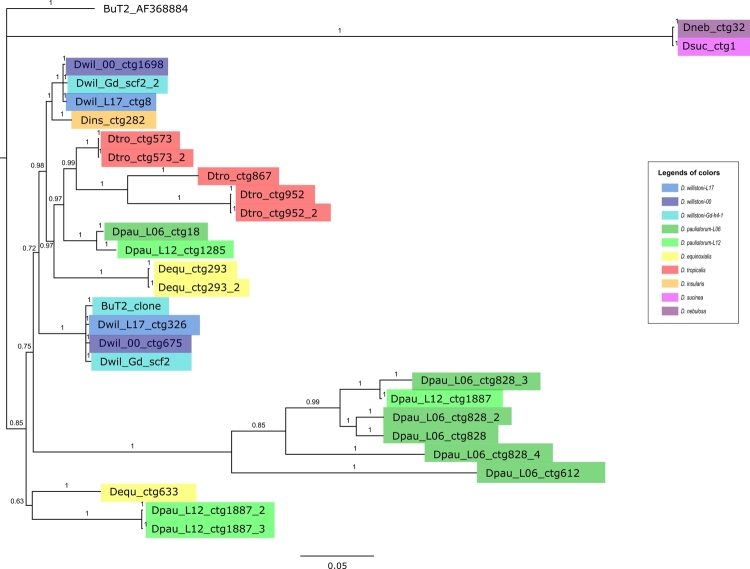
Phylogenetic relationships of the *BuT2* copies in the *willistoni* group. Unrooted Bayesian tree (HKY+G) based on nucleotide sequences. Node supports are shown by posterior probability. *Drosophila buzzatti BuT2* canonical sequence is shown in black; the *BuT2*_clone was used in the FISH experiments. Different strains and species are indicated in different colors, as shown in the legend. Further information about *BuT2* sequences is available in Table S5.

The *BuT2* intragenomic divergence in the different *D. willistoni* strains ranged from 9.11% in *D. willistoni-*Gd-H4-1 and *D. willistoni-*00 to 10.29% in *D. willistoni-*L17. An intragenomic divergence of 13.91% was observed in *D. paulistorum-*L06, and 17.99% in *D. paulistorum-*L12. Interspecies divergence values ranged from 0.13% between *D. nebulosa* and *D. sucinea* to 53.13% between *D. sucinea* and *D. paulistorum-L06*. Table S6 shows the average divergence of the *BuT2* sequences found within and between the species and strains. All copies of *BuT2* retrieved in the sequenced genomes of the *willistoni* group were used to construct a phylogeny (Figure 6). Species of the *bocainensis* and *willistoni* subgroups formed two clusters with well-established relationships. The clade of the *willistoni* subgroup showed different groupings with high probability, with copies of *D. willistoni* strains, *D. paulistorum* strains, and all sequences of *D. tropicalis*. Two groups formed by copies of *D. willistoni* strains showed short branch lengths, which indicates that copies of different strains are very similar. 

*mar* search

We started the search for homologous sequences to the *mar* element in the *willistoni* group genomes by using the query clone_8 from *D. tropicalis* (JQ654772.1), also used in the FISH experiments. *Mar* homologous sequences recovered in the genomes were aligned using the full-length *mar* reconstructed by Deprá et al. (2012) in order to obtain putative full copies.

We recovered *mar*-like sequences in *D. willistoni-*Gd-H4-1, *D. willistoni-*L17, *D. willistoni-*00, *D. paulistorum-*L06, *D. paulistorum-*L12, *D. equinoxialis*, *D. tropicalis*, and *D. insularis* genomes (Figure 3). The exact number of copies in *D. willistoni, D. paulistorum*, and *D. equinoxialis* strains was difficult to determine because the genome contains some small fragmented copies that were not captured in the searches. Also, the copy number is variable among the species. In the *bocainensis* subgroup (*D. sucinea* and *D. nebulosa*) no *mar*-like sequences were identified (Figure 4C). 

*Mar* full-length copies or putatively active were recovered only from the *D. tropicalis* genome. Partially complete copies were observed in *D. willistoni-*Gd-H4-1 (7 copies), *D. willistoni-*L17 (5 copies), and *D. willistoni-*00 (6 copies); degenerate and *mar* MITE copies were identified also in these strains (Figure 3 and Figure 4C). In *D. tropicalis,* we found the most complete sequence (Dtro_ctg748), with 2760 bp but with small gaps, the largest with a 39 bp base at position 2170-2207 in the reconstructed *mar* (Table S7). In the genome of *D. insularis* we recovered only a few copies of *mar* relics (Figure 3 and Figure 4C) and no full-length or MITE. In *D. insularis,* 6 *mar* sequences (Dins_ctg2309_2, Dins_ctg2309_3, Dins_ctg2309_4, Dins_ctg2309_6, Dins_ctg2309_7, and Dins_ctg2309_8) were flanked by the *BEL-LTR* retrotransposon and the *Transib1* transposon (identified by Censor).

*Mar*-MITEs, similarly to canonical *mar* sequences, were retrieved in *D. willistoni-*Gd-H4-1, *D. willistoni-*L17, *D. willistoni-*00, *D. equinoxialis*, and *D. paulistorum*-L12 (Figure 4C). The most degenerate copies were found in *D. paulistorum-*L06 and *D. paulistorum-*L12; in these strains, even the largest sequences had many small deletions. 

For the *mar* divergence analysis, we used the conserved *mar* region in genomes of the *willistoni* subgroup. The intragenomic divergence in the different *D. willistoni* strains varied by around 10.33% in *D. willistoni-*Gd-H4-1, 12.83% in *D. willistoni-*00, and 10.4% in *D. willistoni-*L17 (Table S8). We found an intragenomic divergence of 8.99% in *D. paulistorum*-L06 and 24.51% in *D. paulistorum-*L12. Interspecies divergence ranged from ~17% between the *D. equinoxialis* and *D. willistoni* strains to 43.29% between *D. insularis* and *D. equinoxialis* (Table S8). 

**Figure 7 -:**
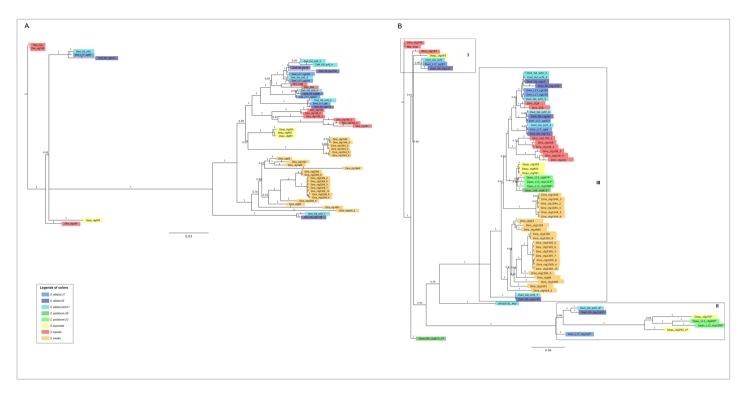
Phylogenetic relationships of the *mar* copies in the *willistoni* subgroup. **A:** Bayesian tree of partially complete *mar* copies in the sequenced genomes of *D. willistoni* strains, *D. equinoxialis, D. tropicalis*, and *D. insularis*. Very degenerate copies of *D. tropicalis* and *D. insularis* were excluded from this analysis. **B**: Bayesian tree of *mar* partially complete, MITEs and relic copies in the sequenced genomes of the *willistoni* subgroup*.* This tree shows partially complete and relic copies used in A, and representative copies of *mar* MITES and degenerate copies in the *D. willistoni* strains*, D. paulistorum* strains*,* and *D. equinoxialis* genomes. AF518731_*mar* is the canonical *mar*-MITE of *D. willistoni*; *mar*_trop was used in the FISH experiments. Three different clades are indicated in boxes I, II, and III. Different strains and species are indicated in different colors, as shown in the legend. Degenerate sequences and MITEs are indicated by asterisks. Further information about *mar* sequences is available in Table S7.

We reconstructed the phylogenetic relationships between *mar* sequences using different methods (for more details see the Material and Methods section) (Figure 7A, 7B, and Figure S2). Figure 7A shows a phylogenetic tree constructed with partially complete sequences obtained from the *willistoni* group genomes, except the degenerate sequences Dtro_ctg838, Dtro_ctg804, and Dins_ctg1175. In Figure 7B, the phylogenetic relationships were generated employing the same sequences from Figure 7A and the representative copies of *mar* MITEs. Degenerate copies were manually selected according to the blocks of the alignments in the genomes of *D. willistoni* (3 strains), *D. paulistorum* (2 strains), and *D. equinoxialis*. In the two phylogenetic reconstructions (Figure 7A and 7B), the potentially complete sequences of *D. tropicalis* were positioned basally in the phylogeny, followed by the partially completed sequences of *D. willistoni*, and degenerate sequences of *D. equinoxialis* (Box I - Fig 7B). *Mar* MITEs and other degenerate sequences (relic sequences) formed a larger cluster composed of a small clade containing two other partially complete sequences of *D. willistoni* (Box II - Fig 7B), and a large clade including the other sequences (Box III - Fig 7B). In box III, sequences from one species usually appeared interspersed among the other species, possibly reflecting a low divergence between some, as well as low posterior probability values. For example, in *D. insularis* there was a clear clustering of the degenerate sequences in one of the well-supported branches; however, some of these sequences are related to MITEs from *D. equinoxialis,* although with low support value (0.57). When analyzing all the sequences recovered in the genomes, it was not possible to clearly identify the relationships established, mainly between MITE and degenerate sequences, probably because of the low sequence divergence (Figure S2).

## Discussion

*Drosophila willistoni* was the first species in the *willistoni* group to be described, by Samuel Williston in 1896 (Dobzhansky and Powell, 1975). Dobzhansky (1950) described the karyotype of the species, and only in 2007 was the first genome sequenced, by the *Drosophila* 12 Genomes Consortium *et al*. (2007). Currently, there are more than 100 *Drosophila* genomes available (Kim et al., 2021). This allows us to carry out more robust analyses to improve knowledge of the mechanisms involved in the evolution of species, transposons, and host genomes. The availability in our laboratory of strains of different geographical origins and which also have their genome sequenced, allows studies such as this one that are important to deepen the knowledge about the differences in the content and distribution of TE in the same species. Here, we conducted a detailed *in-silico* search to analyze *hobo, BuT2*, and *mar* transposons in available genomes of the *willistoni* group (Kim *et al.*, 2021). In addition, we analyzed the copy number and spatial distribution of these *hAT* transposons on polytene chromosomes of some *D. willistoni* strains. Further, *D. willistoni*-Gd-H4-1, *D. willistoni*-WIP-4, and *D. willistoni-*SG12.00 were used for *in-situ* analyses; *D. willistoni-*L17 and *D. willistoni*-00 were used for *in-silico* analysis; and *D. willistoni*-Gd-H4-1 was used for both *in-situ* and *in-silico* analyses. The available genomes were from the *D. willistoni* species subgroup, represented by *D. willistoni, D. paulistorum, D. equinoxialis, D. tropicais*, and *D. insularis*; and the *bocainensis* subgroup, represented by *D. nebulosa* and *D. sucinea*.

Our results showed that the same TEs (*hobo, BuT2*, and *mar*) varied widely in the copy number and structure of copies, even among the different *Drosophila* strains. Regarding the same TEs (Figure 3 and Figure 4A-C), the number of hybridization signals on the polytene chromosomes varied in the different populations: *D. willistoni*-Gd-H4-1, *D. willistoni*-WIP-4, and *D. willistoni*-SG12.00 (Figure 2A-I). Furthermore, in the strains *D. willistoni*-L17, *D. willistoni*-00, and *D. willistoni*-Gd-H4-1, we identified variations in the number and structure of copies of the same TEs (Figure 3 and Figure 4A-C). This suggests that different populations of *D. willistoni* have undergone changes in the TE content or different selective pressures on TE in that host genome. Differences between insertion sites of the same TE in *D. willistoni* strains have been previously observed. Regner et al. (1996) identified by *in-situ* hybridization, in *D. willistoni*-17A2 strain 10 insertion sites of the *P* element coinciding with the breakpoints of inversions, but in *D. willistoni*-WIP-11A observed only hybridization signals on heterochromatin (Regner *et al.*, 1996). Using Southern blot hybridization, Sassi et al. (2005) found differences in the number of TE copies of the *P* element, also among *D. willistoni* populations. In *D. mojavensis*, Palazzo et al. (2014) also found variability in the distribution and number of copies of the *Bari* element in different subspecies. 

We observed different copy numbers for the elements of the *hAT* superfamily in the different *D. willistoni* strains of different subspecies, both *in-situ* and *in-silico*. A similar situation was reported in *D. willistoni-*L17, from an unknown locality in Uruguay, which proved to have many more repetitive fractions, mainly retrotransposons, than *D. willistoni*-00 from Santa Maria de Ostuna, Nicaragua (Kim et al., 2021). The differences observed between the *in-situ* analysis strains, particularly for the *mar* transposon (Figure 1 and Figure 2C, 2F and 2I), may be related to the chromosomal/genomic characteristics of the different populations of the species. *D. willistoni* can be subdivided into three subspecies: *D. w. willistoni*, *D. w. winge*, and *D. w. quechua* (Ayala and Tracey, 1973; Mardiros et al., 2016)*,* that have different geographic distributions. As shown in Figure 1, *D. willistoni* has a predominantly neotropical distribution, from Mexico and south Florida to the southernmost part of South America and from the Pacific to the Atlantic oceans (Spassky et al., 1971; Zanini et al., 2015). The strains used in the *in-situ* and *in-silico* analyses represent populations arranged along the geographic distribution of the different subspecies (Figure 1): *D. willistoni*-Gd-H4-1 (Guadeloupe Island - *willistoni* subspecies), *D. willistoni*-WIP-4 (Bahia, Brazil - *winge* subspecies), and *D. willistoni*-SG12.00 (Montevideo, Uruguay - *winge* subspecies), used in *in-situ* and *in-silico* analyses; and *D. willistoni-L17* (Uruguay - *winge* subspecies) *and D. willistoni-00* (Santa Maria de Ostuna, Nicaragua - *willistoni* subspecies) used only in *in-silico* analyses. 

The differences in copy numbers of the elements of the *hAT* superfamily analyzed here may be related to the chromosomal and genomic plasticity required to allow *D. willistoni* to occupy different habitats within its geographic distribution. The chromosomal and genomic plasticity of *D. willistoni* has been demonstrated in the large number of rearrangements previously found in different populations (Dobzhansky, 1957; Valente and Araújo, 1985; Regner and Valente, 1993; Rohde et al., 2006; Bhutkar et al., 2008; Rohde and Valente, 2012). A characteristic common to all *D. willistoni* populations is paracentric inversions on the five chromosomal arms, although the location and amount of inversions vary among populations - Review in Rohde and Valente (2012). Rohde and Valente (2012) identified and cataloged 50 different rearrangements in 30 populations of polymorphic chromosomes of *D. willistoni* that segregate at different frequencies, with a clear latitudinal cline, from North to South America, along the species’ distribution. 

Additional evidence to support this hypothesis comes from the records of reproductive isolation between strains: populations found in Central America, North America, and northern Caribbean islands are reproductively isolated from South American and southern Caribbean island strains (Figure 1) (Mardiros et al., 2016). Partial reproductive isolation between populations influences gene exchange and consequently influences the differences of transposable elements in different populations.

For *D. willistoni*-Gd-H4-1 (the only one for which we have on our *Drosophila* Laboratory and whole sequenced genome) we obtained different estimates of copy numbers using different approaches (*in-situ* and *in-silico*). Our results showed that with the *hobo* element the different approaches were in accordance with the presence of low copy numbers (one by *in-situ* and three by *in-silico*). For the *BuT2* and *mar* elements, we observed discrepancies between the analyses (Figure 1 and 2). In *hobo*, we found stronger signals (identified by the ImageJ software) and some weaker ones could be seen in the FISH picture (Figure 2A). Also, three *hobo* copies were detected in the sequenced genome, only one of which was complete (Figure 4A). However, in both *BuT2* and *mar*, the number of sequences differed between the two approaches; the largest difference was observed in *mar*, copy number estimated by FISH was higher (by visual analysis) than the number retrieved in the sequenced genome (Figures 1, 2, and 4). The discrepancy between the number of copies found using FISH and *in-silico* may be related to two factors: limitations of each approach and intrinsic characteristics of the *BuT2* and *mar* TEs that make it difficult to identify an absolute number of copies. In the case of *BuT2* and *mar*, elements are considered MITEs, and share structural characteristics such as small nonautonomous elements, present in high and variable copy numbers, conservation of TIRs, and rich in AT region (Bureau and Wessler, 1992; Jiang et al., 2004; Feschotte et al., 2005). Regarding the sequenced genomes, although large amounts of DNA data are available, many genomes are not fully known because of the difficulty in assembling the repetitive fraction, sequences obtained with NGS platforms are short and simply do not span long repetitive sequences, and numerous copies of reads can be nearly identical, leading to the tendency to group them into single and collapsed contigs (Mascher and Stein, 2014; De Bustos et al., 2016). This type of difference has been observed in other studies using different techniques; for instance, in *D. simulans*, with the *hAT hosimary* element, the number of copies estimated by *in-silico* and Southern blot was higher than estimated by FISH (Deprá et al., 2010). Maside et al. (2005) also reported differences between different techniques (PCR amplification and *in-situ* hybridization) of the *S-element* in *D. melanogaster*, noting that the amplification method can be more biased toward high-frequency elements than the *in-situ* method, which uses to identify the insertion sites. 

We also investigated the presence and structure of copies of the *hobo, BuT2*, and *mar* elements in the sequenced genomes of the *willistoni* species group. In our analysis, the *hAT* transposase phylogenetic tree revealed three major clusters of related sequences (Figure S1), as previously referred to as the *Buster* family, *Tip* family, and *Ac* family by Rossato et al. (2014). The *D. willistoni-hobo* putative transposase fell within the *Ac* family, as did the other *hAT* from *Drosophila*, except for the elements *mar* (*Buster* family) and *BuT2* (*Tip* family) (Deprá et al., 2012; Rossato *et al.*, 2014). The *hobo* element TSD consensus sequence (5`-nTnnnnAn-3`) also indicates that *D. willistoni*-*hobo* is an *Ac* element (Arensburger et al., 2011; Rossato *et al.*, 2014). The cluster formed by *D. willistoni*-*hobo* is composed of elements previously described in fly species from different genera: *Drosophila willistoni* (*Howilli2*)*; D. melanogaster* (canonical *hobo*), *D. ananassae* (*Hoana1, Hoana3*, and *Hoana8*), and *D. mojavensis* (Homo1), as well as *Ceratitis capitata* (*Cc-HRE*)*, Bactrocera tryoni* (*Homer*), *Musca domestica* (*Hermes*), and *Lucilia cuprina* (Hermit) (Handler and Gomez, 1996; Ortiz and Loreto, 2009).

*Hobo-like* elements identified in the *willistoni* group genomes are closely related to the canonical *hobo* (*D. melanogaster)*, as conserved and identical TIRs in *D. willistoni* (three sequenced strains), *D. paulistorum* (two sequenced strains), *D. sucinea*, and *D. nebulosa* genomes (Figure 4A-C) (Calvi et al., 1991). However, there was little divergence between the sequences of species in the *willistoni* group, including *D. sucinea* and *D. nebulosa* belonging to the *bocainensis* subgroup. Furthermore, as seen in the phylogenetic tree, the *hobo* copies do not cluster similarly to the phylogeny of the species in the *willistoni* group, so HTT events cannot be ruled out. Moreover, sequences close to *hobo*, called *hobo-brothers* elements, showed incongruities with the TE and host *Drosophila* species phylogenies, suggesting possible cases of horizontal transfer (Bernardo and Loreto, 2013). The presence of *hobo*-like sequences was previously identified only in some strains of *D. willistoni* collected in Brazil, including *D. willistoni*-WIP-4, but were absent in the Amazon strain and in other species of the *willistoni* group, by Southern and Dot blot hybridization (Loreto *et al.*, 1997). In the *melanogaster* subgroup, *hobo* elements were found in three forms: canonical (complete or deleted, lacking the central part of the sequence), relic (having TIRs and conserved subterminal sequences or defective in one TIR), and elements such as MITEs (review by Loreto *et al.*, 2018). We also identified sequences in canonical and relic form in *willistoni* group genomes, except in *D. equinoxialis, D. tropicalis*, and *D. insularis*, since in these genomes we found only degenerate copies (Figure 3). 

*BuT2* and *mar* were characterized as MITE sequences in *D. willistoni* genomes (Holyoake and Kidwell, 2003; Deprá et al., 2012; Rossato et al., 2014). The *BuT2* MITE elements identified in the *D. willistoni*-Gd-H4-1 genome have conserved TIRs but also the unusually low copy number (24 copies) that is common in MITE elements (Rossato *et al.*, 2014). Our *in-silico* searches were not able to recover *BuT2* MITE sequences in genomes of the *willistoni* species group (Figure 3 and Figure 4B). We also identified more *BuT2* hybridization signals in chromosomes of *D. willistoni*-Gd-H4-1 than in the sequenced genome of *D. willistoni*-Gd-H4-1 (Figure 1, Figure 2B and Figure 4B). The likely reason for the differences observed between the *in-silico* and *in-situ* approaches is that our searches retrieved only full-length and relic *BuT2* copies (Figure 3 and Figure 4B). We identified only *BuT2*-like degenerate sequences in the *bocainensis* subgroup, and one fragment each in *D. sucinea* and *D. nebulosa* (Figure 3 and Figure 4B). We found high rates of divergence between the sequences of species from the *willistoni* subgroup and the *bocainensis* subgroup, reaching 53.13% between *D. sucinea* and *D. paulistorum*-L06. This agrees with the phylogenetic tree, which showed the sequences of the *bocainensis* subgroup grouping separately from the *willistoni* subgroup (Table S6 and Figure 5). These sequences of the *bocainensis* subgroup are degenerate copies and have high divergence rates, which may be due either to a stochastic loss of element *BuT2* in the genomes of the *bocainensis* subgroup, or to retrieval of sequences homologous to other *BuT* elements such as *BuT1* in our searches (Cáceres et al., 2001; Wallau et al., 2012). Furthermore, the phylogenetic tree (Figure 5) has *BuT2* copies of the *willistoni* subgroup with a distribution similar to the evolution of the species in the group, and were probably vertically transmitted during the evolution of these species (Rossato *et al.*, 2014; Zanini et al., 2018; Finet et al., 2021). Our results agree with the findings by Rossato *et al.* (2014), who hypothesized that *BuT2* was inserted in the ancestor of the neotropical *willistoni/saltans* groups and that MITEs expanded in the *willistoni* group.

*BuT2* showed more signals of hybridization in *D. willistoni*-Gd-H4-1 and *D. willistoni*-WIP4 (Figure 2B, E), whereas only two hybridization signals were identified in *D. willistoni*-SG12.00 (Figure 2H). Assuming that the many hybridization signals in the *D. willistoni*-Gd-H4-1 chromosome are of the *BuT2* MITE sequences described by Rossato et al. (2014), we hypothesized that the *BuT2* MITE sequences proliferated in *D. willistoni*-Gd-H4-1 and *D. willistoni*-WIP4 but not in *D. willistoni*-SG12.00. The presence of *BuT2* MITE sequences in the *willistoni* group is not completely clear, and further studies with several other strains are necessary. Interestingly, *BuT2* is associated with inversion breakpoints in *D. buzzatii* chromosomes (Cáceres et al., 2001). 

When the *mar* elements were characterized, the only genome of the *willistoni* group sequenced was *D. willistoni*-Gd-H4-1 (Drosophila 12 Genomes Consortium *et al.*, 2007; Deprá et al., 2012). We found *mar* elements only in species of the *willistoni* subgroup (Figure 3 and Figure 4C), reinforcing the idea that this element invaded the genomes after the separation of the *willistoni* and *bocainensis* subgroups, as proposed by Deprá *et al.* (2012), and considering that the *D. willistoni* subgroup diverged approximately 7.3 Mya (review by Zanini et al., 2018).

*Mar* elements were one of the first MITE families discovered in the *D. willistoni* genome (Holyoake and Kidwell, 2003). The origin of the different MITE families is unclear; one hypothesis is that MITEs originate from deletions of autonomous copies (Deprá et al., 2012; Fattash et al., 2013). Only in *D. tropicalis*, a low number of copies and one potentially complete copy (Dtrop_ctg748) were identified (Figure 3 and Figure 4). This sequence is likely ancestral, as apparent from the phylogenetic reconstruction (Figure 7A and 7B). However, in the genomes of *D. willistoni* (three sequenced strains), *D. paulistorum* (two sequenced strains) and *D. equinoxialis* (Figure 3 and Figure 4), we observed expansion of *mar* sequences, possibly originating from deletion of the TE transposase region (Figure 7A and 7B). MITES can be considered genomic superparasites because they conserve the transposase recognition regions for mobilization and are usually found in high copy numbers (Fattash *et al.*, 2013).

TEs in host genomes tend to survive by horizontal transmission to other hosts. When a TE inserts into a new host it tends to proliferation within a genome and within a population, accumulation of mutations, loss of element by inactivation, diversification within host, and element persistence within host (Schaack et al., 2010). In *D. paulistorum*-L06 we found a large number of relic or degenerate copies (Figure 3 and Figure 4), but did not identify MITEs. One hypothesis is that this genome possibly did not undergo expansion of MITEs, but rather of complete copies that mutated over time. Additionally, the lower diversity of the *mar* sequences observed in *D. paulistorum-*L06 (8.99%) compared to the sequences found in *D. paulistorum*-L12 (24.51%) may be a function of the different geographical origins of the strains. *D. paulistorum-*L12 is Andean-Brazilian, from within the large geographic region of origin (Brazil, Ecuador, Peru, Colombia, and Venezuela) (Zanini et al., 2018). *D. paulistorum-*L06 from San Salvador (El Salvador) has been maintained in the laboratory since 1955 (Kim et al., 2021) (Figure 3), explaining the lower diversity of *mar* in this genome. The genome of *D. insularis* retained a low copy number, highly related but relic or degenerate (Figure 3, Figure 4, Figure 7A and 7B). 

The dynamics of the TE and host genome coevolution are complex. In this study we showed the evolutionary history of the elements *hobo*, *BuT2*, and *mar* in the sequenced genomes of the *willistoni* group, as well as the distribution and estimated number of copies in the polytene chromosomes in three strains of *D. willistoni* from different geographic locations. We also compared different approaches (*in-situ* and *in-silico*) in examining the genome of *D. willistoni*-Gd-H4-1. The genome can be viewed as an ecosystem inhabited by diverse communities of TEs that seek to proliferate through interactions with each other TEs and with the genome as a whole and other component of the cell (Venner et al., 2009; Bourque et al., 2018). Evolutionary forces such as natural selection and genetic drift can also shape the distribution and accumulation of TEs in host genomes (Kidwell, 2002; Chénais et al., 2012; Bourque *et al.*, 2018; Moschetti et al., 2020). For example, mobilization in the host genome or colonization of new genomes is necessary to avoid loss by genetic drift, and potentially deleterious inserts will not remain in the population for many generations (Le Rouzic and Capy, 2006; Venner *et al.*, 2009; Bourque *et al.*, 2018). Through a genomic and cytogenetic approach, we reported that different populations (strains) of one species, *D. willistoni*, maintain and share the same transposon differently. Our data also showed that the genetic plasticity enabled by transposable elements can help species such as *D. willistoni* to occupy very different environments over its wide geographic distribution.

## Supplementary material

The following online material is available for this article:

Table S1 - Information for the *Drosophila willistoni* strains used in the FISH experiments.

Table S2 - Information on genomes of the *willistoni* group used in this study.

Table S3 -
*Hobo* sequences identified in the *willistoni* group genomes.

Table S4 - Nucleotide divergence percentages of *hobo* sequences.

Table S5 -
*BuT2* sequences identified in the *willistoni* group genomes.

Table S6 - Nucleotide divergence percentages of *BuT2* sequences.

Table S7 -
*Mar* sequences identified in the *willistoni* group genomes.

Table S8 - Nucleotide divergence percentages of *mar* sequences.

Figure S1 - Phylogenetic relationships of the *hAT* superfamily.

Figure S2 - Neighbor-Joining tree of *mar* sequences.
