# Supplementary material for: Interpopulation variation of transposable elements of the hAT superfamily in Drosophila willistoni (Diptera: Drosophilidae): in-situ approach
Source: Genet Mol Biol. 2022 Mar 16;45(2):e20210287. doi: 10.1590/1678-4685-GMB-2021-0287 (PMC8961557; doi:10.1590/1678-4685-GMB-2021-0287)
Supplement: Table S1 - [file 1415-4757-GMB-45-2-e20210287-s1.pdf]

Genetics and Molecular Biology
https://doi.org/10.1590/1678-4685-GMB-2021-0287

Supplementary material to "Interpopulation variation of transposable elements of the *hAT* superfamily in *Drosophila willistoni* (Diptera: Drosophilidae): *in-situ* approach"

Table S1 - Information for the *Drosophila willistoni* strains used in the FISH experiments.

**Strains of *D. willistoni***	**Geographic location**	**Collection date**	**Geographic coordinates**	**Collected by**
**Gd-H4-1**	Guadeloupe Islands	1991	16°15' N- 61°35' W	J. Powell
**WIP-4**	Ibirapitanga, Bahia, Brazil	1960	12°54' S-38°19' W	Helga Winge and A.R. Cordeiro
**SG12.00**	Montevideo, Uruguay	2000	34°53' S-56°16' W	Beatriz Goñi
