# Supplementary material for: Interpopulation variation of transposable elements of the hAT superfamily in Drosophila willistoni (Diptera: Drosophilidae): in-situ approach
Source: Genet Mol Biol. 2022 Mar 16;45(2):e20210287. doi: 10.1590/1678-4685-GMB-2021-0287 (PMC8961557; doi:10.1590/1678-4685-GMB-2021-0287)
Supplement: Table S2 - [file 1415-4757-GMB-45-2-e20210287-s2.pdf]

Genetics and Molecular Biology
https://doi.org/10.1590/1678-4685-GMB-2021-0287

Supplementary material to "Interpopulation variation of transposable elements of the *hAT* superfamily in *Drosophila willistoni* (Diptera: Drosophilidae): *in-situ* approach"

Table S2 - Species, strains and assemblies of genomes of the *willistoni* group used in this work.

Genus	***Subgenus***	***Group***	***Subgroup***	***Species***	**Strain name[a]**
Drosophila	*Sophophora*	*willistoni*	*willistoni*	*D. willistoni*-L17	L-G3
				D. willistoni-00	NA
				D. willistoni-Gd-H4-1	GD-h4-1
				D. paulistorum-L06	(Heed) H66.1C
				D. paulistorum-L12	L12
				D. equinoxialis	NA
				D. tropicalis	(Heed) H65.2
				D. insularis	jp01i
			bocainensis	*D. sucinea*	49.15
				D. nebulosa	H176.10

[a] Strain names along with corresponding NDSSC stock center numbers were provided by Kim *et. al*. 2021.
