# Supplementary material for: Interpopulation variation of transposable elements of the hAT superfamily in Drosophila willistoni (Diptera: Drosophilidae): in-situ approach
Source: Genet Mol Biol. 2022 Mar 16;45(2):e20210287. doi: 10.1590/1678-4685-GMB-2021-0287 (PMC8961557; doi:10.1590/1678-4685-GMB-2021-0287)
Supplement: Table S3 - [file 1415-4757-GMB-45-2-e20210287-s3.pdf]

Genetics and Molecular Biology
https://doi.org/10.1590/1678-4685-GMB-2021-0287

Supplementary material to "Interpopulation variation of transposable elements of the *hAT* superfamily in *Drosophila willistoni* (Diptera: Drosophilidae): *in-situ* approach"

Table S3 - *Hobo* sequences identified in the *willistoni* group genomes with the nomenclature used in this work, length and the location in the contig or scaffolds.

Genus	***Subgenus***	***Group***	***Subgroup***	***Species***	**Scaffold/contig position**	**Name**
Drosophila	*Sophophora*	*willistoni*	*willistoni*	*D. willistoni*-L17	contig_2:c11570663-11570224	Dwil_L17_ctg2
					contig_116:374204-374643	Dwil_L17_ctg116
					contig_116:1194557-1194841	Dwil_L17_ctg116_2
					contig_8:585969-586408	Dwil_L17_ctg8
					contig_206:264514-264805	Dwil_L17_ctg206
					contig_16:3093225-3099665	Dwil_00_ctg16
				D. willistoni-00	contig_1745:23005-22565	Dwil_00_ctg1745

Genetics and Molecular Biology
https://doi.org/10.1590/1678-4685-GMB-2021-0287

	contig_522:1140-1426	Dwil_00_ctg522
D. willistoni-Gd-H4-1	scf2_1100000004887	Dwil_Gd_scf2
	scf2_1100000011155	Dwil_Gd_scf2_2
	scf2_1100000004854	Dwil_Gd_scf2_3
D. paulistorum-L06	contig_36:c1463801-1463361	Dpau_L06_ctg36
	contig_818:c1823481-1823044	Dpau_L06_ctg818
	contig_405:c154382-153957	Dpau_L06_ctg405
	contig_813:c3203276-3202886	Dpau_L06_ctg813
D. paulistorum-L12	contig_1302:c304983-304542	Dpau_L12_ctg1302
	contig_1696:298949-299389	Dpau_L12_ctg1696
	contig_1502:c92077-91638	Dpau_L12_ctg1502
	contig_1502:83646-84086	Dpau_L12_ctg1502_2

Genetics and Molecular Biology
https://doi.org/10.1590/1678-4685-GMB-2021-0287

	contig_1154:213411-213836	Dpau_L12_ctg1154
D. equinoxialis	contig_758:c2990-2844	Dequ_ctg758
	contig_758:246449-246884	Dequ_ctg758_2
	contig_758:c4177-3882	Dequ_ctg758_3
	contig_178:c78153-71907	Dequ_ctg178
D. tropicalis	contig_87:50052-50492	Dtro_ctg87
	contig_109:271215-271655	Dtro_ctg109
	contig_979:1790162-1790453	Dtro_ctg979
	contig_993:c123966-123575	Dtro_ctg993
	contig_1024:c96591-96280	Dtro_ctg1024
D. insularis	contig_1809:22014-22454	Dins_ctg1809
	contig_1464:c7882-7443	Dins_ctg1464

https://doi.org/10.1590/1678-4685-GMB-2021-0287

		contig_58:c843557-843113	Dins_ctg58
		contig_1021:c8391183813	Dins_ctg1021
bocainensis	*D. sucinea*	contig_141:c7473762-7473322	Dsuc_ctg141
		contig_141:c7913447-7913015	Dsuc_ctg141_2
		contig_4:c4456473-4456041	Dsuc_ctg4
		contig_2:1115483-1115920	Dsuc_ctg2
	D. nebulosa	contig_3:1575774-1582214	Dneb_ctg3
		contig_46:14720546-14720983	Dneb_ctg46
		contig_3:1102167-1102610	Dneb_ctg3_2
		contig_1:c3959237-3958802	Dneb_ctg1
		contig_32:276148462-7615283	Dneb_ctg32

[a] TIRs present only in the 5`-*hobo*
(-) absence

Genetics and Molecular Biology
https://doi.org/10.1590/1678-4685-GMB-2021-0287
