# Supplementary material for: Interpopulation variation of transposable elements of the hAT superfamily in Drosophila willistoni (Diptera: Drosophilidae): in-situ approach
Source: Genet Mol Biol. 2022 Mar 16;45(2):e20210287. doi: 10.1590/1678-4685-GMB-2021-0287 (PMC8961557; doi:10.1590/1678-4685-GMB-2021-0287)
Supplement: Table S4 - [file 1415-4757-GMB-45-2-e20210287-s4.pdf]

Genetics and Molecular Biology
https://doi.org/10.1590/1678-4685-GMB-2021-0287

Supplementary material to "Interpopulation variation of transposable elements of the *hAT* superfamily in *Drosophila willistoni* (Diptera: Drosophilidae): *in-situ* approach"

Table S4 - Nucleotide divergence percentages of *hobo* sequences found within and between species/strains.

Species	***D. willistoni*-Gd-H4-1**	***D. willistoni*-L17**	***D. willistoni*-00**	***D. paulistorum*-L06**	***D. paulistorum*-L12**	***D. equinoxialis***	***D. tropicalis***	***D. insularis***	***D. sucinea***	***D. nebulosa***
D. willistoni-Gd-H4-1	13.88%									
D. willistoni-L17	8.66%	3.91%								
D. willistoni-00	10.51%	7.00%	13.64%							
D. paulistorum-L06	10.19%	5.78%	8.97%	7.98%						
D. paulistorum-L12	8.74%	3.74%	8.04%	5.45%	4.10%					
D. equinoxialis	11.40%	6.84%	7.88%	7.33%	6.43%	4.48%				
D. tropicalis	13.05%	8.35%	9.84%	9.16%	8.33%	8.76%	9.81%			
D. insularis	10.32%	4.65%	7.25%	6.09%	4.71%	6.80%	8.13%	4.39%		
D. sucinea	12.48%	8.31%	10.87%	9.29%	8.67%	7.59%	9.26%	6.79%	11.41%	
D. nebulosa	11.70%	7.17%	10.21%	8.51%	7.43%	7.45%	9.09%	6.33%	8.40%	9.84%
