# Supplementary material for: Interpopulation variation of transposable elements of the hAT superfamily in Drosophila willistoni (Diptera: Drosophilidae): in-situ approach
Source: Genet Mol Biol. 2022 Mar 16;45(2):e20210287. doi: 10.1590/1678-4685-GMB-2021-0287 (PMC8961557; doi:10.1590/1678-4685-GMB-2021-0287)
Supplement: Table S5 - [file 1415-4757-GMB-45-2-e20210287-s5.pdf]

*Genetics and Molecular Biology*
https://doi.org/10.1590/1678-4685-GMB-2021-0287

# Supplementary material to "Interpopulation variation of transposable elements of the *hAT* superfamily in *Drosophila willistoni* (Diptera: Drosophilidae): *in-situ* approach"

**Table S5:** *BuT2* sequences identified in the *willistoni* group genomes with the nomenclature used in this work, length and the location in the contig or scaffolds.

| *Genus* | *Subgenus* | *Group* | *Subgroup* | *Species* | Scaffold/contig position | Name | TIR | Length (bp) | TSD |
|---|---|---|---|---|---|---|---|---|---|
| *Drosophila* | *Sophophora* | *willistoni* | *willistoni* | *D. willistoni*-L17 | contig_8:c956548-950094 | Dwil_L17_ctg8 | cagtgctgccaa | 2695 | ctcccata |
| | | | | | contig_326:10479000-10482557 | Dwil_L17_ctg326 | - | 837 | - |
| | | | | *D. willistoni*-00 | contig_675:c193887-187330 | Dwil_00_ctg675 | cagtgctgccaa [a] | 1032 | gtggttag |
| | | | | | contig_1698:54070-63524 | Dwil_00_ctg1698 | cagtgctgccaa | 2737 | ctcccata |
| | | | | *D. willistoni*-Gd-H4-1 | scf2_1100000004958 | Dwil_Gd_scf2_2 | cagtgctgccaa | 2742 | c(t/a)cccata |
| | | | | | scf2_1100000004967 | Dwil_Gd_scf2 | cagtgctgccaa [a] | 1032 | gtggttag |
| | | | | *D. paulistorum*-L06 | contig_18:c659779-653712 | Dpau_L06_ctg18 | cagtgctgccaa | 1644 | (a/g)gcaaa(a/g)g |
| | | | | | contig_828:33560431-33566507 | Dpau_L06_ctg828 | cagtgctgccaa [a] | 1004 | gctaactg |
| | | | | | contig_828:c33557118-33551042 | Dpau_L06_ctg828_2 | cagtgctgccaa [a] | 1044 | gcagcagc |
| | | | | | contig_828:35734319-35740395 | Dpau_L06_ctg828_3 | cagtgctgccaa [a] | 1025 | gctacctg |
| | | | | | contig_828:c33552056-33545980 | Dpau_L06_ctg828_4 | cagtgctgccaa [a] | 510 | gctacctg |
| | | | | | contig_612:347628-353668 | Dpau_L06_ctg612 | - | 522 | - |
| | | | | *D. paulistorum*-L12 | contig_1887:c16021910-16015834 | Dpau_L12_ctg1887 | cagtgctgccaa [a] | 1042 | gctacctg |
| | | | | | contig_1887:3773899-3780096 | Dpau_L12_ctg1887_2 | - | 1158 | - |
| | | | | | contig_1887:3774096-3780315 | Dpau_L12_ctg1887_3 | - | 1158 | - |
| | | | | | contig_1285:664505-670572 | Dpau_L12_ctg1285 | cagtgctgccaa | 1358 | (a/g)gcaaa(a/g)(a/g) |

| ***Genus*** | ***Subgenus*** | ***Group*** | ***Subgroup*** | ***Species*** | **Scaffold/contig position** | **Name** | **TIR** | **Length (bp)** | **TSD** |
|---|---|---|---|---|---|---|---|---|---|
| | | | | *D. equinoxialis* | contig_633:2034621-2040974 | Dequ_ctg633 | cagtgctgccaa [a] | 1110 | ggttgggga |
| | | | | | contig_293:3234777-3241187 | Dequ_ctg293 | - | 779 | - |
| | | | | | contig_293:32345663240643 | Dequ_ctg293_2 | - | 779 | - |
| | | | | *D. tropicalis* | contig_867:c2136044-2129724 | Dtro_ctg867 | cagtgctgccaa [a] | 998 | - |
| | | | | | contig_952:c423594-417411 | Dtro_ctg952 | cagtgctgccaa [a] | 1215 | ctaacggc |
| | | | | | contig_952:c424174-418097 | Dtro_ctg952_2 | cagtgctgccaa [a] | 1215 | ctaacggc |
| | | | | | contig_573:c1710793-1704667 | Dtro_ctg573 | cagtgctgccaa | 2184 | ggcggagc |
| | | | | | contig_573:c1710825-1704789 | Dtro_ctg573_2 | cagtgctgccaa | 2184 | ggcggagc |
| | | | | *D. insularis* | contig_282:21964482-202902 | Dins_ctg282 | cagtgctgccaa | 2546 | - |
| | | | *bocainensis* | *D. sucinea* | contig_1:26154067-26163191 | Dsuc_ctg1 | - | 764 | - |
| | | | | *D. nebulosa* | contig_32:25989124-25995248 | Dneb_ctg32 | - | 764 | - |

[a] TIRs present only in the 3`-*BuT2*

(-) absence
