# Supplementary material for: Interpopulation variation of transposable elements of the hAT superfamily in Drosophila willistoni (Diptera: Drosophilidae): in-situ approach
Source: Genet Mol Biol. 2022 Mar 16;45(2):e20210287. doi: 10.1590/1678-4685-GMB-2021-0287 (PMC8961557; doi:10.1590/1678-4685-GMB-2021-0287)
Supplement: Table S6 - [file 1415-4757-GMB-45-2-e20210287-s6.pdf]

Genetics and Molecular Biology
https://doi.org/10.1590/1678-4685-GMB-2021-0287

Supplementary material to "Interpopulation variation of transposable elements of the *hAT* superfamily in *Drosophila willistoni* (Diptera: Drosophilidae): *in-situ* approach"

Table S6 - Nucleotide divergence percentages of *BuT2* sequences found within and between species/strains.

Species	***D. willistoni*-Gd-H4-1**	***D. willistoni*-L17**	***D. willistoni*-00**	***D. paulistorum*-L06**	***D. paulistorum*-L12**	***D. equinoxialis***	***D. tropicalis***	***D. insularis***	***D. sucinea***	***D. nebulosa***
D. willistoni-Gd-H4-1	9.11%									
D. willistoni-L17	5.03%	10.29%								
D. willistoni-00	4.74%	5.01%	9.11%							
D. paulistorum-L06	22.29%	23.77%	22.11%	13.91%						
D. paulistorum-L12	14.15%	14.36%	13.98%	17.90%	17.99%					
D. equinoxialis	10.00%	9.48%	10.01%	25.68%	16.38%	7.20%				
D. tropicalis	13.20%	15.02%	13.11%	17.20%	15.63%	15.40%	5.30%			
D. insularis	5.88%	6.59%	5.77%	17.98%	12.61%	9.81%	10.04%			
D. sucinea	24.50%	24.63%	24.83%	53.13%	35.92%	33.75%	37.34%	25.99%		
D. nebulosa	24.70%	24.83%	25.02%	53.02%	35.98%	33.97%	37.46%	26.12%	0.13%	
