# Supplementary material for: Interpopulation variation of transposable elements of the hAT superfamily in Drosophila willistoni (Diptera: Drosophilidae): in-situ approach
Source: Genet Mol Biol. 2022 Mar 16;45(2):e20210287. doi: 10.1590/1678-4685-GMB-2021-0287 (PMC8961557; doi:10.1590/1678-4685-GMB-2021-0287)
Supplement: Table S7 - [file 1415-4757-GMB-45-2-e20210287-s7.pdf]

Genetics and Molecular Biology
https://doi.org/10.1590/1678-4685-GMB-2021-0287

Supplementary material to "Interpopulation variation of transposable elements of the *hAT* superfamily in *Drosophila willistoni* (Diptera: Drosophilidae): *in-situ* approach"

Table S7 - *Mar* sequences identified in the *willistoni* group genomes with the nomenclature used in this work, length and the location in the contig or scaffolds.

Genus	*Subgenus*	*Group*	*Subgroup*	*Species*	Scaffold/contig position	Name	TIR	Length (bp)	TSD
Drosophila	*Sophophora*	*willistoni*	*willistoni*	*D. willistoni*-L17	contig_229:c66505-74287	Dwil_L17_cgt229	cagggcccggc	2341	gtcta(g/t)tt
					contig_97:c2133965-2140917	Dwil_L17_cgt97	caggggtaggc	2260	ctctac(t/c)c
					contig_326:c7390880-7398393	Dwil_L17_cgt326	cagggcccggca [a]	2030	ct(c/g)t(a/c)(c/a)(a/t)a
					contig_8:c742956-735649	Dwil_L17_cgt8	-	1724	-
					contig_227:c32390491-32398000	Dwil_L17_cgt227	cagggcccggc [a]	1998	gattagaac
					contig_169: c265415-266051	Dwil_L17_ctg169	-	562	-
				D. willistoni-00	contig_713:c8475-1168	Dwil_00_ctg713	-	1754	-
					contig_137:c334978-328626	Dwil_00_cgt137	gtccggcaatt [a]	1382	-
					contig_1745:c162134-155401	Dwil_00_ctg1745	gcttaacttgt [a]	1644	-
					contig_881:c1057949-1065188	Dwil_00_ctg881	-	1797	-
					contig_1102:c1-1993	Dwil_00_ctg1102	caggggccgac [a]	1069	ctctacaa
					contig_10:c138987-131462	Dwil_00_ctg10	caggggccggc [a]	1998	ctctacaa/actggagt
					contig_1263:c81211-81822	Dwil_00_ctg1263	-	519	-
				D. willistoni-Gd-H4-1	scf2_1100000004958:c721448-728037	Dwil_Gd_scf2	caggggtaggc	2268	ctctac(t/c)c
					scf2_1100000004958:c2486062-2478755	Dwil_Gd_scf2_2	-	1722	ttcgatga [b]
					scf2_1100000012712 :c1372-1	Dwil_Gd_scf2_3	-	1319	-
					scf2_1100000004967:c4275204-4267679	Dwil_Gd_scf2_4	tcccaagcggc [a]	2006	tta aga gt/tta ttc ga
					scf2_1100000011190:c1676-1	Dwil_Gd_cf2_5	-	1495	-
					scf2_1100000004884:c190698-183177	Dwil_Gd_scf2_6	-	1927	-
					scf2_1100000004542:c67336-60603	Dwil_Gd_scf2_7	tcaggacgaat [a]	1701	attctatg [b]
					scf2_1100000004830:c439968-439226	Dwil_Gd_scf2_8	-	646	-
				D. paulistorum-L06	contig_813:c 2236897-2235283	Dpau_L06_ctg813	-	1512	-
					contig_813:c 2180492-2179440	Dpau_L06_ctg813_2	-	897	-
				D. paulistorum-L12	contig_674:c188482-186568	Dpau_L12_ctg674	-	1517	-
					contig_1323:c12301-10250	Dpau_L12_ctg1323	-	1777	-
					contig_1886:c769625-767711	Dpau_L12_ctg1886	-	1516	-

Genus	*Subgenus*	*Group*	*Subgroup*	*Species*	Scaffold/contig position	Name	TIR	Length (bp)	TSD
					contig_990:c45776-44933	Dpau_L12_ctg990	-	497	-
					contig_1590:c386924-386165	Dpau_L12_ctg1590	-	458	-
				D. equinoxialis	contig_583:c1253850-1246431	Dequ_ctg583	caggggtaggc [a]	1530	-
					contig_293:c869557-861796	Dequ_ctg293	cagggcccggc [a]	1639	-
					contig_991:c95981-89067	Dequ_ctg991	cagggcccggc [a]	1075	-
					contig_483c:259082-266841	Dequ_ctg483	cagggcccggc [a]	1640	-
					contig_270c:154589-155437	Dequ_ctg270	-	655	-
					contig_293:c1072823-1073495	Dequ_ctg293_2	-	523	-
				D. tropicalis	contig_748:c123411-114941	Dtro_ctg748	cagaggtagac	2760	not conserved
					contig_197:16059-24004	Dtro_ctg197	caggggtaggc [a]	2068	-
					contig_9:586622-594136	Dtro_ctg9	cagggcccggc [a]	1966	-
					contig_258:749883-757356	Dtro_ctg258	-	1934	-
					contig_108:c303354-295980	Dtro_ctg108	cagggcccg [a]	1867	-
					contig_108:48387-55723	Dtro_ctg108_2	cagggcccg [a]	1849	-
					contig_108:c467912-464549	Dtro_ctg108_3	caggggccggc [a]	473	-
					contig_108:191753-198105	Dtro_ctg108_4	caggggccggc [a]	476	-
					contig_838:c27084-20915	Dtro_ctg838	-	984	-
					contig_1190:30021-30178	Dtro_ctg1190	caggggtaggc [a]	2033	-
					contig_804:1-3233	Dtro_ctg804	-	333	-
					contig_191:c27800-23642	Dtro_ctg191	caggggccggc [a]	474	-
				D. insularis	contig_1865:1562-6147	Dins_ctg1865	cagggcccggc [a]	1657	-
					contig_1203:17415-24357	Dins_ctg1203	cagggcccggc [a]	1110	-
					contig_2309:37451-44372	Dins_ctg2309	cagggcccggc [a]	2019	-
					contig_2309:3201-9992	Dins_ctg2309_2	cagggcccggc [a]	1175	-
					contig_2309:7064-13855	Dins_ctg2309_3	cagggcccggc [a]	1175	-
					contig_2309:10926-17717	Dins_ctg2309_4	cagggcccggc [a]	1175	-
					contig_2309:20253-26866	Dins_ctg2309_5	cagggcccgcc	769	-
					contig_2309:4000-10443	Dins_ctg2309_6	cagggcccggc [a]	1175	-
					contig_2309:7863-14306	Dins_ctg2309_7	cagggcccggc [a]	1175	-
					contig_2309:11725-18168	Dins_ctg2309_8	cagggcccggc [a]	1175	-
					contig_2309:38380-44823	Dins_ctg2309_9	cagggcccggc [a]	2019	-

https://doi.org/10.1590/1678-4685-GMB-2021-0287

Genus	***Subgenus***	***Group***	***Subgroup***	***Species***	**Scaffold/contig position**	**Name**	**TIR**	**Length (bp)**	**TSD**
					contig_2309:136-7443	Dins_ctg2309_10	-	1003	-
					contig_1691:54802-61944	Dins_ctg1691	-	1171	-
					contig_424:c512443-505582	Dins_ctg424	-	1409	-
					contig_1044:75760-82098	Dins_ctg1044	-	1509	-
					contig_1044:88090-93428	Dins_ctg1044_2	-	1508	-
					contig_1044:76350-82617	Dins_ctg1044_3	-	1509	-
					contig_1044:88680-93947	Dins_ctg1044_4	-	1271	-
					contig_1044:c148079-141744	Dins_ctg1044_5	-	1508	-
					contig_1044:c146789-140520	Dins_ctg1044_6	-	1486	-
					contig_1175:27855-33891	Dins_ctg1175	-	728	-
					contig_63:c394755-393231	Dins_ctg63	cagggcccggc [a]	2099	-
					contig_1948:c3029-1043	Dins_ctg1948	cagggcccggc [a]	813	-
					contig_68:c8004-787	Dins_ctg68	cagggcccggc [a]	1610	-

[a] TIRs present only in the 5`-*mar*

[b] Information of several hits representing *D. willistoni* strains, *D. paulistorum* strains, and *D. equinoxialis mar*-MITE and degenerated copies were omitted from this table, except sequences used in the phylogenetic tree in Figure 7.

(-) absence
