# Supplementary material for: Interpopulation variation of transposable elements of the hAT superfamily in Drosophila willistoni (Diptera: Drosophilidae): in-situ approach
Source: Genet Mol Biol. 2022 Mar 16;45(2):e20210287. doi: 10.1590/1678-4685-GMB-2021-0287 (PMC8961557; doi:10.1590/1678-4685-GMB-2021-0287)
Supplement: Table S8 - [file 1415-4757-GMB-45-2-e20210287-s8.pdf]

Genetics and Molecular Biology
https://doi.org/10.1590/1678-4685-GMB-2021-0287

Supplementary material to "Interpopulation variation of transposable elements of the *hAT* superfamily in *Drosophila willistoni* (Diptera: Drosophilidae): *in-situ* approach"

Table S8 - Nucleotide divergence percentages of *mar* sequences found within and between species/strains.

Species	*D. willistoni*-Gd-H4-1	*D. willistoni*-L17	*D. willistoni*-00	*D. paulistorum*-L06	*D. paulistorum*-L12	*D. equinoxialis*	*D. tropicalis*	*D. insularis*
D. willistoni-Gd-H4-1	10.33%							
D. willistoni-L17	10.30%	10.40%						
D. willistoni-00	11.53%	11.59%	12.83%					
D. paulistorum-L06	31.72%	32.03%	31.82%	8.99%				
D. paulistorum-L12	22.63%	22.56%	23.57%	23.51%	24.51%			
D. equinoxialis	17.83%	17.54%	17.54%	38.74%	27.48%	20.91%		
D. tropicalis	31.18%	30.80%	30.99%	24.91%	31.37%	40.42%	22.02%	
D. insularis	35.06%	35.79%	35.61%	20.78%	32.27%	43.29%	19.96%	10.49%
