# Supplementary material for: Interpopulation variation of transposable elements of the hAT superfamily in Drosophila willistoni (Diptera: Drosophilidae): in-situ approach
Source: Genet Mol Biol. 2022 Mar 16;45(2):e20210287. doi: 10.1590/1678-4685-GMB-2021-0287 (PMC8961557; doi:10.1590/1678-4685-GMB-2021-0287)
Supplement: Figure S1 - [file 1415-4757-GMB-45-2-e20210287-s9.pdf]

Genetics and Molecular Biology
https://doi.org/10.1590/1678-4685-GMB-2021-0287

Supplementary material to "Interpopulation variation of transposable elements of the *hAT* superfamily in *Drosophila willistoni* (Diptera: Drosophilidae): *in-situ* approach"

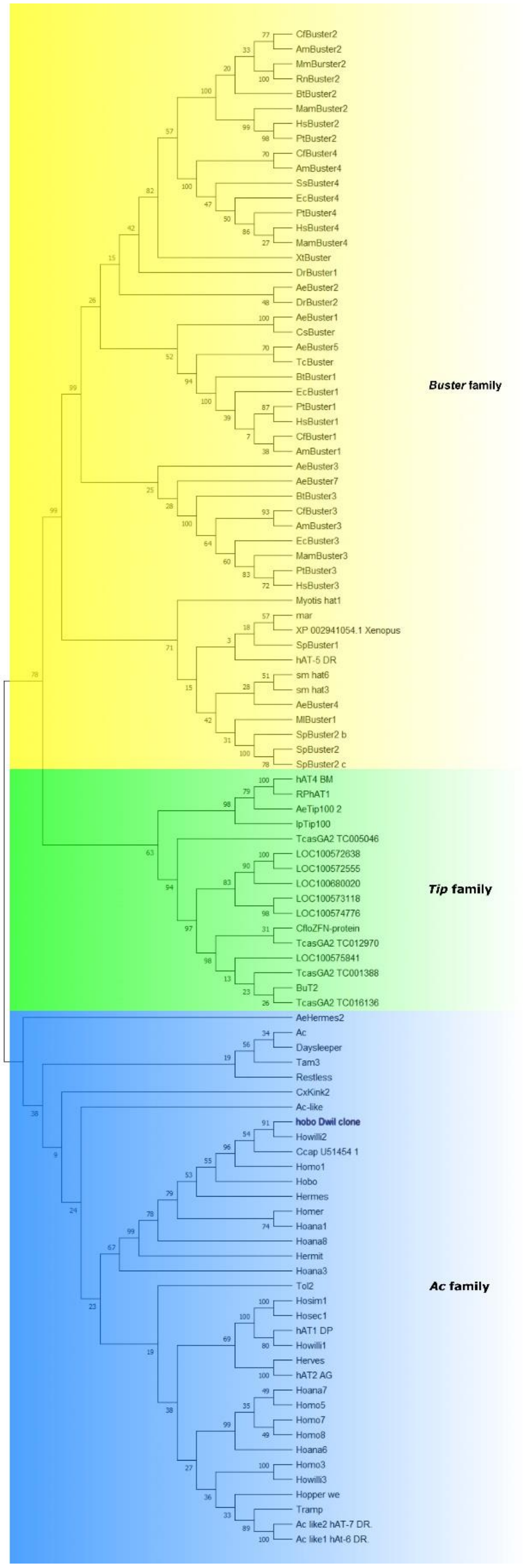

https://doi.org/10.1590/1678-4685-GMB-2021-0287

Figure S1 - Phylogenetic relationships of the *hAT* superfamily. Unrooted phylogenetic tree of *hAT* element amino-acid transposase sequences by maximum likelihood, using MEGA X and the Le-Gascuel model (Le and Gascuel 2008; Kumar et al. 2018). Clade colors denote the different families of *hATs* reported by Rossato et al. (2014). Bootstrap values of nodes are percentages for 1000 replicates.
