# Supplementary material for: Interpopulation variation of transposable elements of the hAT superfamily in Drosophila willistoni (Diptera: Drosophilidae): in-situ approach
Source: Genet Mol Biol. 2022 Mar 16;45(2):e20210287. doi: 10.1590/1678-4685-GMB-2021-0287 (PMC8961557; doi:10.1590/1678-4685-GMB-2021-0287)
Supplement: Figure S2 - [file 1415-4757-GMB-45-2-e20210287-s10.pdf]

Genetics and Molecular Biology
https://doi.org/10.1590/1678-4685-GMB-2021-0287

Supplementary material to "Interpopulation variation of transposable elements of the *hAT* superfamily in *Drosophila willistoni* (Diptera: Drosophilidae): *in-situ* approach"

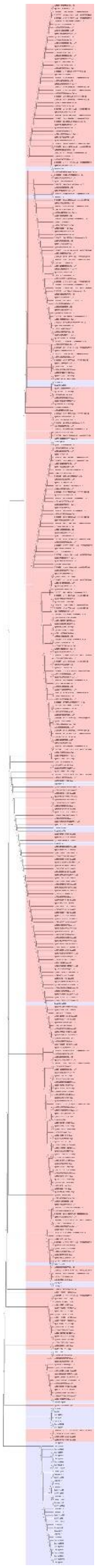

https://doi.org/10.1590/1678-4685-GMB-2021-0287

Figure S2 - Neighbor-Joining tree all partially complete *mar*, MITES, and relic copies. Identical, small, and/or very degenerate copies of each genome were excluded from this analysis (see Material and Methods). Node supports are bootstrap values (1000 replicates). Sequences used in Figure 7A and 7B are highlighted in blue, and other sequences are highlighted in red, except Dwil_Gd_scf2_3; Dins_ctg2309_5, Dins_ctg424, Dins_ctg1175, Dins_ctg1948; Dtro_ctg108_3, Dtro_ctg108_4, Dtro_ctg838, Dtro_ctg804, Dtro_ctg191.
